# Tumor‐Adhesive Chitosan‐Derived Multi‐Immune Agonist Unleashes Strong and Durable Anti‐Cancer Immunity

**DOI:** 10.1002/advs.202414110

**Published:** 2025-02-25

**Authors:** Huilan He, Liang Liu, Yun Zheng, Jinlong Ji, Li Cao, Chunlian Ye, Yu Sun, Ying Zhang, Zhiyuan Zhong

**Affiliations:** ^1^ College of Pharmaceutical Sciences and State Key Laboratory of Radiation Medicine and Protection Soochow University Suzhou 215123 China; ^2^ Biomedical Polymers Laboratory College of Chemistry, Chemical Engineering and Materials Science Soochow University Suzhou 215123 China; ^3^ School of Optical and Electronic Information Suzhou City University Suzhou 215104 China

**Keywords:** cancer therapy, immune agonist, immunomodulation, multi‐targets, radiotherapy

## Abstract

The immunomodulation of the tumor microenvironment is critical for effective cancer immunotherapy, particularly for tumors that exhibit limited responses to conventional treatments. However, current immune agonists developed for tumor immunomodulation face several challenges, such as poor intratumoral retention, inadequate biocompatibility, and restricted cellular targets, which ultimately hamper their therapeutic efficacy and clinical application. In this study, a tumor‐adhesive chitosan‐tethered immune agonist construct (TACTIC) is introduced, which demonstrates good biocompatibility and robust immunostimulatory effects, enhancing the immunogenicity of tumor cells while simultaneously stimulating pro‐inflammatory responses in various immune cell populations. Mechanistic investigations reveal that TACTIC targets multiple signaling pathways, conferring it to effectively remodel the irradiated tumor microenvironment, improve tumor control on murine cancer models post‐radiotherapy, and elicit systemic immune responses with memory effects. The findings highlight the potential of TACTIC as a powerful macromolecular immune adjuvant, paving the way for its broader application in innovative cancer immunotherapies.

## Graphical abstract

A tumor‐adhesive chitosan‐derived immune agonist TACTIC is developed for strong and durable anti‐tumor immunity. The immune agonist exhibits multi‐targets and modulates both tumor cell immunogenicity and pro‐inflammatory responses in immune cells, thereby potentiating the therapeutic efficacy of clinical radiotherapy.

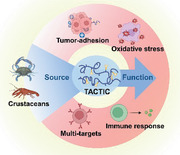

## 1 Introduction

Cancer immunotherapies have emerged as a promising approach for the clinical treatment of solid tumors by enabling the host immune system to specifically recognize and eliminate tumor cells.^[^ ^1^, ^2^ ^]^ In recent years, conventional cancer treatment modalities, such as radiotherapy (RT) and chemotherapy, have been recognized for their ability to initiate anti‐cancer immune responses.^[^ ^3^, ^4^ ^]^ However, these treatments generally result in suboptimal immune activation due to the insufficient induction of pro‐inflammatory signals, thereby diminishing the overall therapeutic efficacy.^[^ ^5^, ^6^ ^]^ To overcome this limitation, a variety of immune agonists that are capable of enhancing immune stimulation by targeting specific cellular signaling pathways have been developed.^[^ ^7^, ^8^ ^]^ Despite these progresses, most of immune agonists with small molecular architecture still face significant challenges in clinical translation, particularly due to issues such as rapid systemic clearance, poor intratumoral retention, and undesirable off‐target immune toxicities.^[^ ^9^, ^10^, ^11^ ^]^ These limitations highlight the urgent need for more effective immune modulators that can achieve sustained therapeutic effects while minimizing adverse effects.

Recently, macromolecular immune agonists have gained substantial attention in the field of cancer immunotherapy.^[^ ^12^, ^13^, ^14^, ^15^ ^]^ Compared to their small molecular counterparts, macromolecular immune agonists possess the capacity to engage in multivalent interactions with cellular receptors and achieve prolonged retention in tumor microenvironment, thereby eliciting more potent and sustained immune responses.^[^ ^16^, ^17^, ^18^ ^]^ However, the poor biocompatibility and the limited biodegradability of the polymer backbones employed in the macromolecular immune agonists remain significant barriers to their clinal translation.^[^ ^19^, ^20^ ^]^ Moreover, current macromolecular immune agonists are generally restricted to modulating a single immune signaling pathway, potentially limiting their ability to comprehensively reprogram the tumor microenvironment and elicit a diversified immune response. Additionally, given that tumor cells are the predominant cell type in solid tumors, particularly for the immunologically “cold” tumors, developing macromolecular immune agonists capable of simultaneously enhancing tumor cell immunogenicity and stimulating pro‐inflammatory responses in immune cells is crucial to achieve robust modulation of the tumor microenvironment and amplify adaptive anti‐cancer immunity.

**Figure 1:**
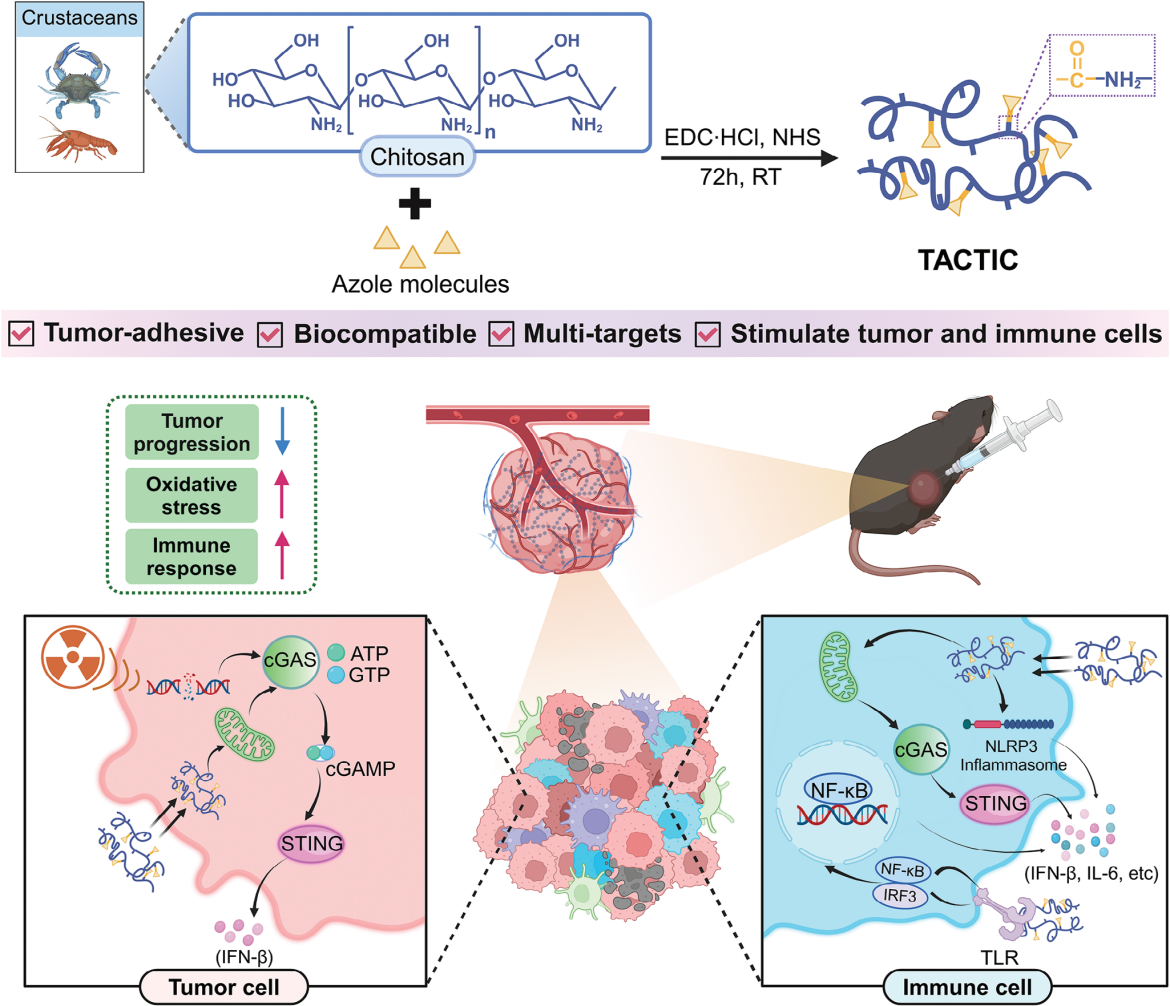
Overview of the preparation of TACTIC and its mechanism of action in immune modulation. TACTIC exhibits tumor adhesion and good biocompatibility, enhances the immunogenicity of tumor cells, and simultaneously promotes pro‐inflammatory responses in immune cells, thereby offering the promising potential to achieve robust cancer therapeutic effects in conjugation with RT. This figure was created with Biorender.com.

In this study, we report the development of a tumor‐adhesive chitosan‐tethered immune agonist construct (TACTIC) for achieving strong and durable anticancer immunity. Through a facile and scalable technique, TACTIC was engineered with multi‐targeted immunostimulatory properties capable of simultaneously enhancing tumor cell immunogenicity and stimulating pro‐inflammatory responses in immune cells, thereby promoting a synergistic enhancement of anti‐cancer immunity post‐RT (**Figure** **1**). In vivo studies demonstrated that TACTIC exhibited prolonged tumor retention and significantly remodeled the irradiated tumor microenvironment, resulting in the suppression of tumor growth on murine tumor models and prolonged survival of mice. Our findings present a potent tumor‐adhesive macromolecular immune agonist with multi‐targets and high therapeutic potential, providing a new paradigm for the design of next‐generation cancer immunotherapies.

## 2 Results

### 2.1 TACTIC Potently Augments the Immunogenicity of Tumor Cells

**Figure 2:**
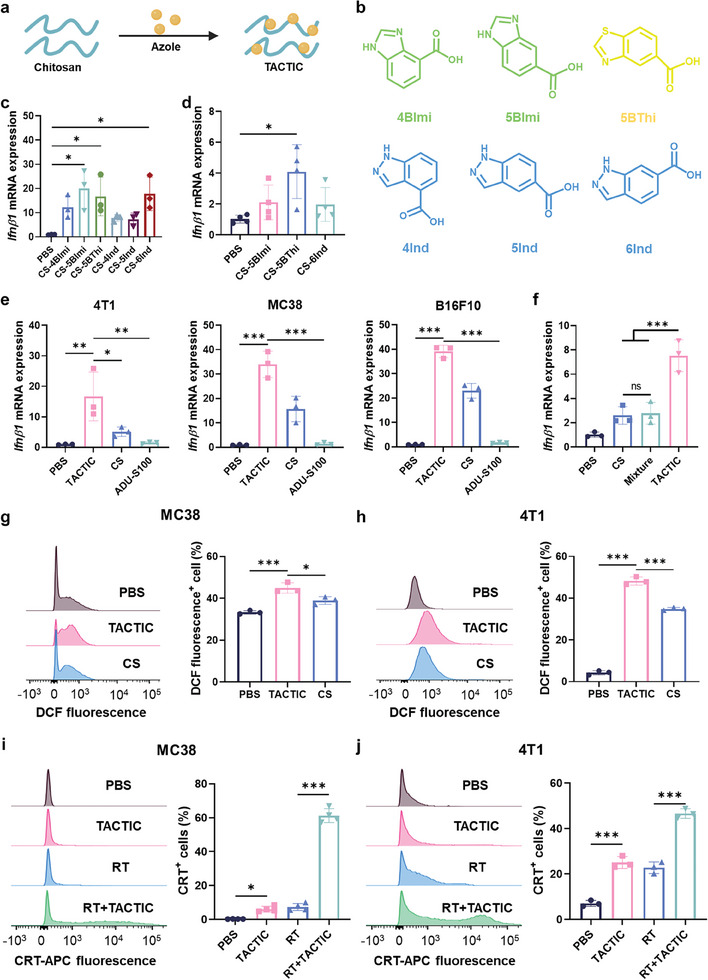
a) A brief illustration of the preparation of TACTIC. b) Chemical structures of different azole molecules used in this study. c) The relative mRNA expression of *Ifnβ1* in 4T1 cells after treatment with TACTICs (*n* = 3). d) The relative mRNA expression of *Ifnβ1* in RAW264.7 macrophages after treatment with TACTICs (*n* = 4). e) The relative mRNA expression of *Ifnβ1* in 4T1 cells, MC38 cells and B16F10 cells after treatment with TACTIC, CS or ADU‐S100 (*n* = 3). f) The relative mRNA expression of *Ifnβ1* in 4T1 cells after treatment with CS, TACTIC, or the mixture of CS with 5BThi molecules. The concentrations of CS and 5BThi molecules were equivalent with those in TACTIC (*n* = 3). g) Histogram graphs of DCF fluorescence in MC38 cells and the percentages of DCF fluorescence positive MC38 cells after treatment with TACTIC or CS (*n* = 3). h) Histogram graphs of DCF fluorescence in 4T1 cells and percentages of DCF fluorescence positive 4T1 cells after treatment with TACTIC or CS (*n* = 3). i) Histogram graphs of CRT expression in MC38 cells and the percentages of CRT positive MC38 cells after treatment with TACTIC, RT, or their combination (*n* = 4). j) Histogram graphs of CRT expression in 4T1 cells and the percentages of CRT positive 4T1 cells after treatment with TACTIC, RT, or their combination (*n* = 3). Statistical significance was calculated via one‐way ANOVA test in c–j. *p < 0.05, **p < 0.01, ***p < 0.001.

We aimed to develop a tumor‐adhesive macromolecular immune agonist with good biocompatibility to simultaneously enhance tumor cell immunogenicity and stimulate pro‐inflammatory responses in immune cells, thereby priming a robust anti‐cancer immune response. To achieve this, we utilized the U.S. Food and Drug Administration (FDA)‐approved polysaccharide chitosan (CS) and conjugated it with azole compounds commonly found in anti‐fungal agents with capacities to generate reactive oxygen species (ROS).^[^ ^21^, ^22^, ^23^ ^]^ In this study, a range of azole compounds, including 4BImi (benzoimidazole‐4‐carboxylic acid), 5BImi (benzoimidazole‐5‐carboxylic acid), 5BThi (benzothiazole‐5‐carboxylic acid), 4Ind (indazole‐4‐carboxylic acid), 5Ind (indazole‐5‐carboxylic acid) and 6Ind (indazole‐6‐carboxylic acid), have been selected and investigated for their effects in orchestrating a potent TACTIC (**Figure** **2**a,b; Figures S1–S5 and Table S1, Supporting Information).

The cyclic GMP‐AMP synthase (cGAS)‐stimulator of interferon genes (STING) pathway is a key component of the innate immune response, responsible for sensing DNA from invading pathogens or double‐stranded DNA released from damaged nuclear or mitochondrial compartments.^[^ ^24^, ^25^ ^]^ Upon activation, cGAMP is synthesized and binds to STING proteins in the endoplasmic reticulum, triggering STING translocation to the Golgi apparatus and activating TBK1 (TANK‐binding kinase 1) and IRF3 (interferon regulatory factor 3) to drive the production of type‐I interferons (IFN‐Is).^[^ ^26^, ^27^ ^]^ Given the wide expression of the cGAS‐STING signaling pathway in mammalian cells, we first evaluated whether TACTICs that were synthesized by covalently conjugating azole molecules onto CS could stimulate cGAS‐STING activation in both tumor cells and immune cells. By assessing the induction of IFN‐I response, we found that CS‐5BImi, CS‐5BThi, and CS‐6Ind can effectively upregulate the expression of *Ifnβ1*, a key marker of cGAS‐STING activation, in 4T1 breast tumor cells (Figure 2c). CS‐5BThi was further identified as the optimal TACTIC for subsequent studies due to its strongest effects in priming an IFN‐I response in RAW264.7 macrophages (Figure 2d). Analyses revealed that the commercially available cGAS‐STING agonist ADU‐S100 and the small molecular tether 5BThi were relatively ineffective in inducing an IFN‐I response in tumor cells (Figure 2e; Figure S6, Supporting Information). In contrast, the covalent conjugation of 5BThi significantly enhanced the efficacy of CS in upregulating the expression of *Ifnβ1* across several tumor cell lines, including 4T1 breast tumor cells, MC38 colorectal cancer cells, and B16F10 melanoma cells (Figure 2e). Importantly, the robust IFN‐I response triggered by TACTIC could not be replicated by simple mixing of CS and 5BThi, highlighting the significance of covalent conjugation for TACTIC in enhancing the immunogenicity of tumor cells (Figure 2f). Notably, TACTIC exhibited effective cellular interaction and negligible cytotoxicity at the concentrations tested (Figures S7–S9, Supporting Information).

We then evaluated the cellular ROS production induced by TACTIC. Treatment with 5BThi alone did not significantly increase ROS levels in either MC38 colorectal cancer cells or 4T1 breast tumor cells (Figure S10, Supporting Information). However, as demonstrated in Figure 2g,h, TACTIC, synthesized by covalently conjugating 5BThi to CS, exhibited a significantly greater capacity to induce cellular ROS production than unmodified CS. This trend was consistent with the levels of DNA damage induced by 5BThi, CS, and TACTIC (Figure S11, Supporting Information). Damaged DNA is a key trigger for cGAS‐STING signaling pathway, which subsequently initiates an IFN‐I response. Therefore, the enhanced ability of TACTIC to induce an IFN‐I response may be associated with its increased efficacy in promoting cellular ROS production and inducing DNA damage, suggesting its promising potential for priming multifaceted immunostimulatory effects. Tumor‐derived lactate is a key metabolite contributing to the immunosuppressive microenvironment of solid tumors.^[^ ^28^ ^]^ STING activation has been reported to inhibit aerobic glycolysis in tumor cells, thereby reducing lactate secretion in a manner independent of innate immunity.^[^ ^29^ ^]^ Consistent with these findings, both TACTIC and CS were shown to suppress the secretion of lactate from tumor cells (Figure S12, Supporting Information), indicating their potential to mitigate tumor‐induced immunosuppression. Furthermore, we investigated the impact of TACTIC on the immunogenic cell death (ICD) of tumor cells. TACTIC alone effectively induced significant ICD in both MC38 colorectal cancer cells and 4T1 breast tumor cells, with these effects further potentiated when combined with RT (Figure 2i,j).

### 2.2 TACTIC Effectively Activates Immune Cells via Multi‐Targets

**Figure 3:**
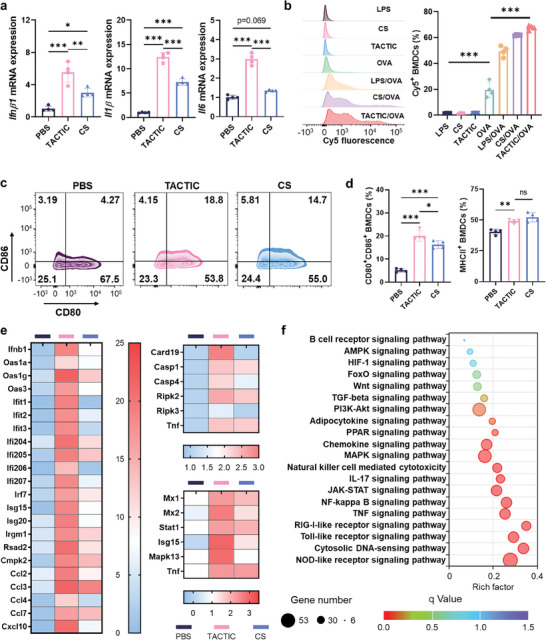
a) The relative mRNA expression of *Ifnβ1*, *Il1β*, and *Il6* in RAW264.7 macrophages after treatment with CS or TACTIC (*n* = 4). b) Histograms of Cy5 positive cells in BMDCs after treatment with LPS, CS, TACTIC, naked OVA‐Cy5, the pre‐complexation of LPS/Cy5‐OVA, CS/Cy5‐OVA or TACTIC/Cy5‐OVA (*n* = 4). c) Representative flow cytometry analyses of the activation of BMDCs after treatment with CS or TACTIC. d) Percentages of CD80^+^CD86^+^ cells or MHCII^+^ cells among BMDCs after treatment with CS or TACTIC (*n* = 4). e,f) RNA sequencing analyses of BMDCs after treatment with CS or TACTIC. e) Heatmaps of the expression of different genes after treatment. f) KEGG analyses of differentially expressed genes (DEGs) in BMDCs. Statistical significance was calculated via one‐way ANOVA test in a, b, and d. *p < 0.05, **p < 0.01, ***p < 0.001.

Tumor‐associated macrophages (TAMs) are a predominant innate immune population within solid tumors and play pivotal roles in modulating the tumor immune microenvironment.^[^ ^30^ ^]^ Therefore, understanding how TACTIC affects the immune response in these cells is vital for assessing its potential in cancer immunotherapy. We evaluated the effects of TACTIC on inducing pro‐inflammatory responses in RAW264.7 macrophages. Although 5BThi was ineffective in inducing pro‐inflammatory responses in these cells, its conjugation on CS significantly rendered the obtained TACTIC with superior effects compared to CS in priming the expression of multiple pro‐inflammatory cytokines, including *Ifnβ1*, *Il1β*, and *Il6* (**Figure** **3**a; Figure S13, Supporting Information).

Dendritic cells (DCs) are the most proficient antigen‐presenting cells (APCs) and play a crucial role in antigen presentation, which is essential for initiating a tumor‐specific adaptive immune response.^[^ ^31^ ^]^ To investigate how unmodified CS and TACTIC influences these processes of DCs, we isolated mouse bone marrow‐derived dendritic cells (BMDCs) and treated them in vitro with a pre‐complex of lipopolysaccharide (LPS, as a positive control)/Cy5‐labeled ovalbumin (OVA, a model tumor antigen), CS/Cy5‐OVA or TACTIC/Cy5‐OVA. As shown in Figure 3b, the internalization of OVA alone was observed to be moderate. Similar to LPS, CS and TACTIC significantly enhanced OVA uptake by DCs. Further analyses revealed that TACTIC exhibited greater capacities than CS in promoting the activation of DCs, leading to higher proportions of CD80^+^CD86^+^ cells and comparable proportions of MHCII^+^ cells among CD11c^+^ DCs (Figure 3c,d). To gain deeper insights into the molecular pathways through which TACTIC stimulates the innate immune response, we conducted RNA sequencing to analyze changes in gene expression following treatment. Heatmap analyses revealed a significant upregulation of genes associated with the cGAS‐STING pathway (e.g., *Ifnβ1*, *Oas3*, and *Cxcl10*) and NOD‐like receptors (NLRs) (e.g., *Casp1*, *Ripk2*) in BMDCs treated with TACTIC compared to untreated controls or those treated with CS alone (Figure 3e).^[^ ^32^, ^33^, ^34^ ^]^ Enrichment analyses of various signaling pathways demonstrated that the immunostimulatory effects of TACTIC extend beyond the cGAS‐STING pathway and NOD‐like receptors; they also involved other cellular signaling mechanisms, including Toll‐like receptors and RIG‐1 like receptors (Figure 3f). This multifaceted targeting by TACTIC likely contributes to its robust immunostimulatory effects.

These findings indicate that TACTIC not only modulates tumor cells but also exerts significant immunomodulatory effects on key immune cells within the tumor microenvironment. By enhancing the immunogenicity of tumor cells, stimulating the pro‐inflammatory response in macrophages, and promoting antigen uptake and maturation in DCs, TACTIC has the potential to reshape the tumor immune microenvironment in favor of a more effective anti‐cancer immune response.

### 2.3 TACTIC Exhibits Heterogeneous Cellular Distribution, Prolonged Tumor Retention, and Considerable Anti‐Cancer Efficacy In Vivo

**Figure 4:**
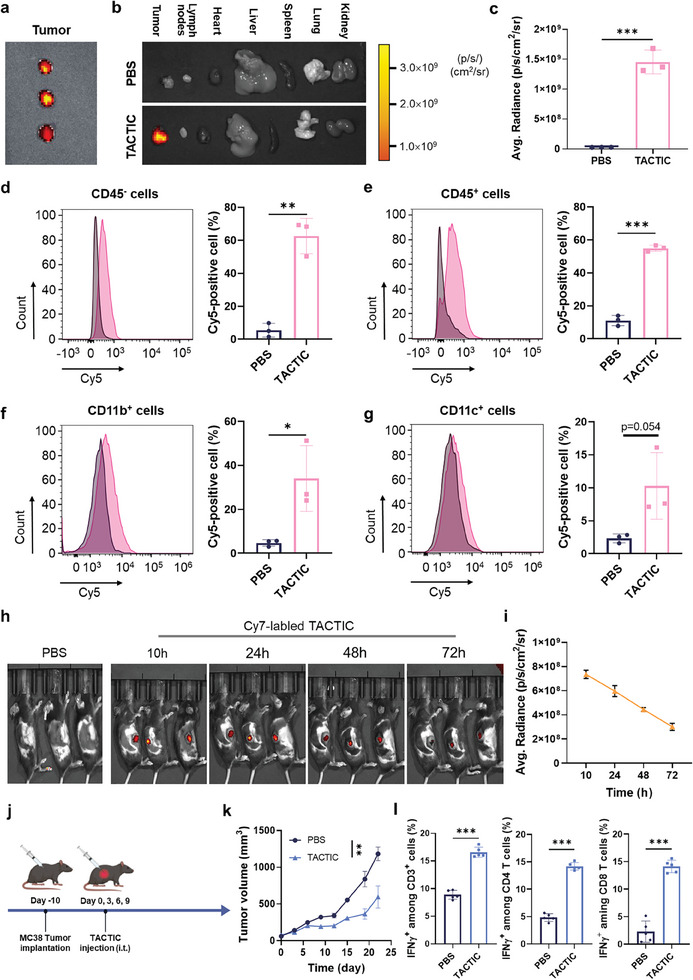
a) IVIS images of MC38 tumor tissues isolated from Cy5‐labeled TACTIC injected tumor‐bearing mice. b) IVIS images of MC38 tumor tissues, TDLNs, and major organs (heart, liver, spleen, lung, and kidney) isolated from PBS or Cy5‐labeled TACTIC injected tumor‐bearing mice. c) Quantification of Cy5 fluorescent intensity in MC38 tumor tissues isolated from Cy5‐labeled TACTIC injected tumor‐bearing mice (*n* = 3). Histogram graphs of Cy5 fluorescence and percentages of Cy5‐positive cells among d) CD45^−^ cells, e) CD45^+^ cells, f) CD45^+^CD11b^+^ cells, and g) CD45^+^CD11c^+^ cells in MC38 tumor tissues (*n* = 3), (black lines: PBS; pink lines: TACTIC treatment). h) IVIS images of the whole body of MC38 tumor‐bearing mice at indicated time points and i) quantification of Cy7 fluorescent intensity in tumor sites after Cy7‐labeled TACTIC was intratumorally injected (*n* = 3). j) Scheme for the anti‐cancer studies on MC38 tumor model. k) Average tumor growth curves of MC38 tumors after indicated treatment (*n* = 6). l) Percentages of IFNγ^+^ cells among CD3^+^ T cells, CD4 (CD3^+^CD4^+^) T cells, and CD8 (CD3^+^CD8^+^) T cells in the spleens of mice after indicated treatment (*n* = 5). For a–g, tumor tissues, TDLNs, and organs were isolated from mice at 24 h post‐administration. i.t.: Intratumoral. Statistical significance was calculated via unpaired *t*‐test in c–g and k,l. *p < 0.05, **p < 0.01, ***p < 0.001.

Intratumoral injection is a clinically relevant approach that minimizes the systemic side effects associated with cancer therapeutics and has been approved by the FDA for certain cancer treatments, such as oncolytic virus therapy for melanoma.^[^ ^35^ ^]^ To investigate the biodistribution of TACTIC in vivo, we established murine MC38 colorectal cancer model and administrated TACTIC intratumorally. Prior to injection, TACTIC was labeled with Cy5 for fluorescent imaging and tracking. As shown in **Figure** **4**a,c, TACTIC predominantly accumulated within tumor tissues at 24 h post‐injection, with minimal fluorescent signals detected in the tumor‐draining lymph nodes (TDLNs) and major organs, including the heart, liver, spleen, lung, and kidney. To further analyze the cellular distribution of TACTIC within the tumor microenvironment, we prepared single‐cell suspensions from tumor tissues and stained the cells with antibodies specific to various cell subtypes. The data revealed that TACTIC was internalized by both CD45^−^ tumor/stroma cells and CD45^+^ immune cells (Figure 4d,e). Further analyses of Cy5 fluorescence within immune cell subtypes indicated the effective uptake of TACTIC by CD45^+^CD11b^+^ myeloid cells and CD45^+^CD11c^+^ DCs (Figure 4f,g). Given the previously demonstrated robust immunostimulatory effects of TACTIC on tumor cells, macrophages, and DCs (Figures 2 and 3), this heterogeneous cellular distribution of TACTIC within solid tumors following injection is a promising indicator of its potential to modulate the tumor microenvironment and elicit considerable anti‐cancer performance in vivo.

To evaluate the retention of TACTIC within tumor tissues after intratumoral injection, we examined its presence at different time points post‐injection. TACTIC was labeled with Cy7 for imaging. Notably, strong Cy7 fluorescent signals were still observed at the tumor sites three days post‐injection of Cy7‐labeled TACTIC, with ≈40% of the fluorescent intensity measured at 10 h post‐injection (Figure 4h,i). This indicates the remarkable tumor retention of TACTIC and suggests that a three‐day interval for intratumoral injections could be optimal for maintaining its high concentrations within tumor tissues, thereby sustaining robust modulation of the tumor microenvironment and enhancing the efficacy of cancer immunotherapy. Subsequently, we investigated the therapeutic effects of intratumorally injected TACTIC on MC38 colorectal cancer‐bearing mice (Figure 4j). The results demonstrated that TACTIC alone significantly suppressed MC38 tumor growth, highlighting its potential as an effective adjuvanted therapeutic agent in vivo (Figure 4k). Further analyses of the splenocytes from the treated mice revealed a marked increase in the percentages of IFNγ^+^ T cells compared to those from untreated control mice, indicating a robust anti‐cancer immune response induced by the local TACTIC treatment (Figure 4l).

### 2.4 TACTIC Elicits Remarkable Therapeutic Effects on Focally Irradiated Cancer‐Bearing Mice

**Figure 5:**
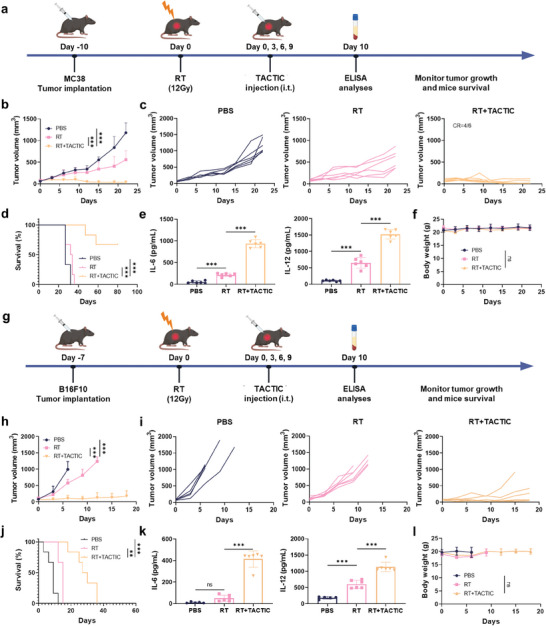
a) Scheme for the anti‐cancer studies on MC38 colorectal cancer‐bearing mice. b) Average tumor growth curves after indicated treatment. c) Individual tumor growth curves in b). d) Survival rate of mice after indicated treatment. e) Concentrations of IL‐6 and IL‐12 in the serum of MC38 colorectal cancer‐bearing mice after indicated treatments. f) Average body weight of mice after indicated treatment (*n* = 6). g) Scheme for the anti‐cancer studies on B16F10 melanoma‐bearing mice. h) Average tumor growth curves after indicated treatment. i) Individual tumor growth curves in h). j) Survival rate of mice after indicated treatment. k) Concentrations of IL‐6 and IL‐12 in the serum of B16F10 melanoma‐bearing mice after indicated treatments. l) Average body weight of mice after indicated treatment (PBS: *n* = 5; others: *n* = 6). i.t.: Intratumoral. CR: Complete response. Statistical significance was calculated via one‐way ANOVA test in b, e,f, h, and k,l, and the log‐rank test in d and j. *p < 0.05, **p < 0.01, ***p < 0.001.

RT is one of the most widely utilized cancer treatment modalities in clinical practice.^[^ ^36^, ^37^ ^]^ However, the anti‐cancer responses induced by RT alone are generally insufficient for durable tumor suppression, necessitating the incorporation of immunostimulatory agents to achieve robust in situ vaccination effects for clinically significant therapeutic outcomes.^[^ ^38^, ^39^ ^]^ In this context, we investigated the potential of intratumorally administrated TACTIC to enhance the therapeutic efficacy of RT and whether this combination elicits a robust immune response in vivo (**Figure** **5**a). As illustrated in Figure 5b–d, TACTIC substantially improved the tumor response to RT, leading to prolonged survival in MC38 colorectal cancer‐bearing mice, with four out of six mice remaining disease‐free after treatment. Serum cytokine analyses revealed a significant increase in IL‐6 and IL‐12 concentrations in mice treated with the RT + TACTIC combination compared to those receiving RT alone (Figure 5e). Importantly, no significant changes were observed in the body weight of the mice during treatment, indicating the good biocompatibility of TACTIC (Figure 5f).

An essential characteristic of an in situ vaccine is its broad applicability across diverse tumor types, independent of specific tumor antigen expression.^[^ ^40^, ^41^ ^]^ To examine the generalizability of the RT + TACTIC treatment, we established aggressive syngeneic murine models of B16F10 melanoma and performed the local RT + TACTIC treatments on these mice (Figure 5g). Similar to our findings in the MC38 colorectal cancer model, RT alone produced a modest delay in the growth of B16F10 melanoma (Figure 5h‐i). However, when combined with the intratumoral injection of TACTIC, the therapeutic response was significantly amplified, resulting in prolonged overall mice survival (Figure 5h–j). At day 10 post‐initiation of treatment, serum levels of both IL‐6 and IL‐12 were significantly elevated in mice treated with the RT + TACTIC combination compared to control and irradiated groups (Figure 5k). Throughout the study, no significant changes were observed in the body weight of the mice (Figure 5l).

### 2.5 TACTIC Primes a Robust and Systemic Anti‐Cancer Immune Response with Memory Effects

**Figure 6:**
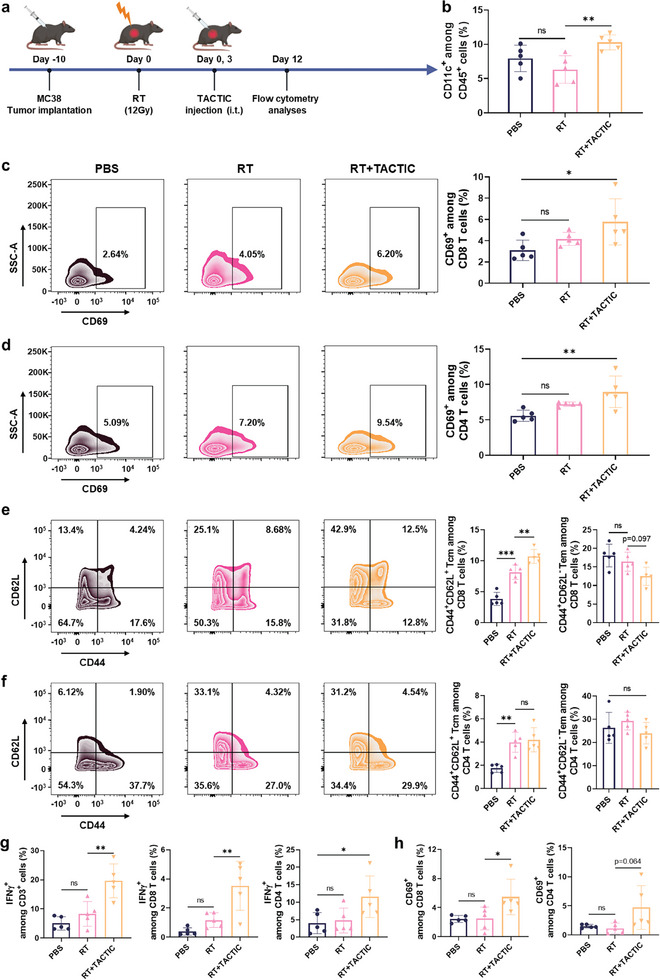
a) Scheme for the studies. b) Percentages of CD11c^+^ DCs among CD45^+^ cells in tumor tissues. Representative flow cytometry analyses and percentages of CD69^+^ cells among c) CD3^+^CD8^+^ T cells and d) CD3^+^CD4^+^ T cells in TDLNs. Representative flow cytometry analyses and percentages of CD44^+^CD62L^+^ Tcm and CD44^+^CD62L^−^ Tem among e) CD3^+^CD8^+^ T cells and f) CD3^+^CD4^+^ T cells in spleens. g) Percentages of IFNγ^+^ cells among CD3^+^ T cells, CD3^+^CD8^+^ T cells, and CD3^+^CD4^+^ T cells in blood. h) Percentages of CD69^+^ cells among CD3^+^CD8^+^ T cells and CD3^+^CD4^+^ T cells in blood. (*n* = 5) i.t.: Intratumoral. Statistical significance was calculated via one‐way ANOVA test in b–h. *p < 0.05, **p < 0.01, ***p < 0.001.

To investigate whether local RT + TACTIC treatment modulated the tumor immune microenvironment and induced an adaptive anti‐cancer immune response, we intratumorally administrated TACTIC into the irradiated MC38 colorectal cancer‐bearing mice and collected tumors and TDLNs at day 12 post‐initiation of treatment (**Figure** **6**a). Flow cytometry analyses were performed to study the changes in immune cells post‐treatment. The data as shown in Figure 6b and Figure S14 (Supporting Information) demonstrated that the injection of TACTIC significantly increased the infiltration of CD11c^+^ DCs into irradiated tumors, while the proportions of CD3^+^ T cells and CD11b^+^ myeloid cells among CD45^+^ immune cells remained unchanged at this time point. These may indicate that TACTIC promoted DC recruitment through pro‐inflammatory signaling and chemokine induction, but does not immediately alter other immune cell populations in the tumor microenvironment. Subsequently, we analyzed the TDLNs, critical lymphoid organs for priming adaptive immunity, and revealed that RT + TACTIC treatment promoted higher expression of CD69, an early activation marker, on both CD3^+^CD8^+^ and CD3^+^CD4^+^ T cells (Figure 6c,d). These results suggested that the DCs infiltrated in tumors also drove the activation of T cells in TDLNs. Together, these findings indicated that local RT + TACTIC treatment effectively elicited an adaptive anti‐tumor immune response.

To assess whether the local RT + TACTIC treatment elicited a systemic immune response with memory effects, we collected the splenocytes and blood cells from the mice. By analyzing the memory T cells in the spleens, we found that local RT + TACTIC treatment resulted in an increased percentage of CD44^+^CD62L^+^ central memory T cells (Tcm) within both CD3^+^CD4^+^ T cell and CD3^+^CD8^+^ T cell populations in secondary lymphoid organs (Figure 6e,f). While a reduction in CD44^+^CD62L^−^ effector memory T cells (Tem) was observed among CD3^+^CD8^+^ T cells following RT + TACTIC treatment, their levels among CD3^+^CD4^+^ T cells remained relatively unchanged (Figure 6e,f), likely due to the dynamics of circulating Tem.^[^ ^42^ ^]^ We then analyzed the cytotoxicity marker (IFNγ) and activation marker (CD69) on circulating T cells to evaluate the systemic immune response induction post‐treatment. We found RT treatment alone had minimal effects on these markers, whereas the local RT + TACTIC combination treatment significantly increased the populations of cytotoxic and activated T cells in the blood, suggesting the generation of a systemic immune response (Figure 6g,h). Histological analyses revealed negligible toxic effects of local RT + TACTIC treatment on major normal tissues, including heart, liver, spleen, lung, and kidney (Figure S15, Supporting Information). These findings suggest that local RT + TACTIC treatment not only modulates the tumor microenvironment but also initiates a systemic anti‐cancer immune response with memory effects, which are critical for effective in situ cancer vaccination.

## 3 Discussion

The urgent need for tumor‐adhesive immune agonists capable of inducing durable and potent immunomodulation within the tumor microenvironment is critical for achieving effective anti‐cancer responses. In this study, we explored the TACTIC strategy to enhance anti‐cancer immunotherapy. Derived from CS, an FDA‐approved polysaccharide known for its good biocompatibility and biodegradability, TACTIC was designed to possess multi‐targeted immunostimulatory capacities and modulatory effects on both tumor cells and immune cells.

In recent years, there is a growing interest in macromolecular immune agonists due to their multi‐valent interactions with targeted receptors and their potential for prolonged retention in tumors.^[^ ^43^, ^44^ ^]^ For instance, Gao et al. identified a cyclic seven‐membered ring tethered polymer (PC7A) from a polymer library that induced robust immune responses, surpassing the efficacy of commercially available immune agonists such as alum and lipopolysaccharide (LPS). Mechanism studies suggested that the robust immune activity of PC7A was due to its direct binding to STING proteins.^[^ ^16^ ^]^ Similarly, Chen et al. conjugated a series of azole molecules on branched polyethylenimine (PEI) to enable it with capacities of STING pathway stimulation.^[^ ^19^ ^]^ Moreover, Hubbel et al. developed a macromolecular immune agonist p(Man‐TLR7) that contains monomers to target DCs via mannose‐binding receptors and stimulate DCs via TLR7 activation. p(Man‐TLR7) was revealed with a higher potency in both innate and adaptive immune stimulation than its small molecular counterparts.^[^ ^45^ ^]^ However, a significant limitation for these macromolecular immune agonists is their non‐degradable backbones, which pose substantial barriers to clinical translation.^[^ ^11^ ^]^ By employing CS as the framework for TACTIC, we addressed this concern, leveraging its biocompatibility and biodegradability.

Another challenge faced by current immune agonists is their limited targeting, generally focused on single signaling pathways. This restriction can hinder their capacity to elicit comprehensive immune responses, especially in cases where targeted pathways may mutate, leading to immune evasion. In contrast, our TACTIC strategy demonstrated the ability to engage multiple cellular signaling pathways, including cGAS‐STING, NOD‐like receptors, Toll‐like receptors, and RIG‐I‐like receptors, as confirmed by RNA sequencing analyses. This multi‐targeted approach holds great promise for generating diverse and sustained immune responses. Furthermore, by effectively modulating both tumor cells and immune cells, TACTIC enhances tumor immunogenicity while stimulating immune responses, highlighting its promise in cancer immunotherapy.

In vitro investigations revealed the multi‐targets and diversified immunomodulatory effects of TACTIC across different cell types (Figures 2 and 3). Notably, TACTIC exhibited tumor‐adhesive properties, remaining within tumor tissues for at least three days, surpassing the retention times of some commercially available small molecular immune agonists (Figure 4).^[^ ^10^ ^]^ The heterogeneous distribution of TACTIC within solid tumors is critical for its immunomodulatory efficacy. Subsequent investigations into the anti‐cancer effects of TACTIC revealed significant inhibition of MC38 colorectal cancer growth (Figure 4). Importantly, when combined with clinical RT, TACTIC markedly enhanced anti‐cancer efficacy, resulting in multiple mice tumor‐free post‐treatment. The effectiveness of TACTIC in enhancing the responsiveness of solid tumors to RT was also observed in aggressive murine cancer models, such as B16F10 melanomas (Figure 5), suggesting the broad applicability of this approach across diverse solid tumor models. Immune cell analyses further indicated the generation of systemic immune responses with memory effects following TACTIC and RT combination treatment (Figure 6).

In future studies, it would be important to further optimize the azole molecules conjugated to CS for achieving more robust multifaceted immunostimulatory effects. Parameters such as the molecular weight and degree of deacetylation of CS, as well as the grafting rate of azole molecules, are also essential for fine‐tuning the immunostimulatory efficacy of TACTIC. In the current work, we focused on TACTIC as an immune adjuvant in cancer immunotherapy, and more comprehensive investigations into the mechanisms underlying its multifaceted immunostimulatory effects will be valuable in future studies. Additionally, it would be valuable to investigate the potential benefits of TACTIC injection with less frequency or TACTIC injection before radiotherapy in future studies, which may offer additional insights into optimizing this approach. Moreover, although intratumoral injection has been FDA‐approved for certain cancer treatments, future research should explore modifications to TACTIC that facilitate safe and effective systemic delivery.

Despite these limitations, our findings provide compelling evidence that TACTIC is powerful in tumor immunomodulation to facilitate robust cancer immunotherapy. This strategy represents a promising translational approach for the development of macromolecular immune agonists in cancer immunotherapies.

## 4 Conclusion

In summary, we developed TACTIC with good biocompatibility and multi‐targeted immunostimulatory effects for enhancing the immunogenicity of tumor cells while simultaneously stimulating immune cells. Our findings demonstrated that the intratumoral administration of TACTIC effectively reprogramed the microenvironment of irradiated solid tumors, leading to a robust anti‐cancer immune response. Notably, the combination of RT and TACTIC treatment resulted in ≈67% of mice complete remission from MC38 colorectal cancer, while significantly inhibiting the growth of aggressive B16F10 melanomas. These compelling results highlight the potential of TACTIC as a powerful platform for modulating the tumor microenvironment and augmenting the efficacy of clinical RT. Given the promising preclinical outcomes, TACTIC warrants further exploration in clinical settings, where its ability to enhance anti‐cancer immunity may revolutionize current cancer treatment paradigms.

## 5 Experimental Section

### Materials

Chitosan (degree of deacetylation >95%) was obtained from Macklin. 1‐Ethyl‐3‐(3‐dimethylaminopropyl) carbodiimide hydrochloride (EDC·HCl) was purchased from Aladdin. N‐hydroxysuccinimide (NHS) was purchased from TCI. Benzoimidazole‐4‐carboxylic acid, benzoimidazole‐5‐carboxylic acid, benzothiazole‐5‐carboxylic acid, indazole‐4‐carboxylic acid, indazole‐5‐carboxylic acid, and indazole‐6‐carboxylic acid were purchased from InnoChem Science & Technology. ADU‐S100 (disodium salt) was purchased from MedChemExpress. The Reactive Oxygen Species Assay Kit and DNA Damage Assay Kit by γH2AX Immunofluorescence were purchased from Beyotime Biotechnology. Calcein‐AM/PI Live/Dead Cell Double Stain Kit was purchased from Solarbio. Mouse ELISA kits (IL‐6 and IL‐12p70) were purchased from Thermo Fisher Scientific. HiScript lll RT SuperMix for qPCR (+gDNA wiper) and Taq Pro Universal SYBR qPCR Master Mix were purchased from Vazyme. The sequences of primers used for RT‐qPCR and the antibodies used for flow cytometry are detailed in Tables S2 and S3 (Supporting Information).

### Cell Lines and Tumor Models

MC38 and 4T1 cell lines were obtained from the National Collection of Authenticated Cell Cultures and maintained in RPMI 1640 medium (Gibco) supplemented with 10% fetal bovine serum (FBS) and 1% penicillin/streptomycin. RAW264.7 macrophages were purchased from Procell (Wuhan, China) and cultured in RAW264.7 cell‐specific culture medium (Procell). B16F10 cells were acquired from the American Type Culture Collection (ATCC) and cultured in DMEM (high glucose, BasalMedia) containing 10% FBS and 1% penicillin/streptomycin. All cell cultures were maintained at 37 °C in a humidified incubator with 5% CO_2_.

To generate bone marrow‐derived dendritic cells (BMDCs), mouse bone marrow was harvested from C57BL/6 mice, and bone marrow cells were cultured in RPMI 1640 medium supplemented with 10% FBS, 1% penicillin/streptomycin, 0.1% 2‐mercaptoethanol and 20 ng mL^−1^ recombinant mouse GM‐CSF (rmGM‐CSF) for 7 days. All cell cultures were maintained at 37 °C in a humidified incubator with 5% CO_2_.

Female C57BL/6 mice (6–8 weeks) were purchased from the Shanghai Laboratory Animal Center (Shanghai, China). All animal experiments were approved by the Animal Care and Use Committee of Soochow University, and all protocols conformed to the Guide for the Care and Use of Laboratory Animals (approval number: SUDA20240716A03).

To establish tumor‐bearing mice, C57BL/6 female mice were intradermally engrafted with tumor cells (MC38 colorectal cancer model: 1 × 10^6^ cells on the right flank; B16F10 melanoma model: 1 × 10^5^ cells on the right flank). Once the volumes of tumors reached 60–100 mm^3^, mice were randomized and treatment was begun.

### Synthesis and Characterization of TACTICs

TACTICs were synthesized by using 1‐ethyl‐3‐(3‐dimethylaminopropyl) carbodiimide (EDC·HCl) and N‐hydroxysuccinimide (NHS) as condensing agents to form amide bonds between carboxyl groups of azole molecules and amino groups of chitosan. Briefly, chitosan (6.2 mmol, 1 g) was dissolved in deionized water by adding 0.1 mol L^−1^ HCl solution, and then the solution was adjusted to pH 5.50 with 0.1 mol L^−1^ NaOH solution. EDC·HCl (2.33 mmol), NHS (3.10 mmol), and azole molecules (e.g., benzothiazole‐5‐carboxylic acid, 1.55 mmol) were dissolved in dimethyl sulfoxide (DMSO) and reacted at 37 °C for 8 h, followed by transferring into the chitosan solution and stirring at room temperature for 72 h. During the reaction, the pH of the solution was adjusted to 6.0–6.5 using 0.1 mol L^−1^ NaOH solution. After dialysis against the mixture of DMSO and deionized water, the solution was further dialyzed against deionized water and the TACTICs product was obtained by lyophilization. The grafting rates of TACTICs were determined by a UV‐vis spectrophotometer (Thermo Scientific).

### Cytotoxicity Assay

The viability of cells treated with TACTIC was evaluated using the Cell Counting Kit‐8 (CCK‐8) assay and the Calcein‐AM/PI Live/Dead Cell Double Stain assay. For the CCK‐8 assay, MC38 or RAW264.7 cells were seeded in 96‐well plates at a density of 3 × 10^3^ cells per well. 293T cells were seeded in 96‐well plates at a density of 5 × 10^3^ cells per well. Following overnight incubation, the cells were treated with TACTIC solutions at different concentrations. After incubating for another 24 h, the culture medium was carefully removed, and 100 µL of fresh medium was added to each well. Subsequently, CCK‐8 reagent (1/10, v/v) was added to the medium and incubated for 1 h. The absorbance at 450 nm was then measured using a microplate reader to evaluate the cells viability. For the Calcein‐AM/PI Live/Dead Cell Double Stain assay, MC38 cells were seeded in 12‐well plates at a density of 2 × 10^5^ cells per well. Following overnight incubation, the cells were treated with 5BThi, TACTIC, or CS (CS: 60 µg mL^−1^). After incubating for another 24 h, the cells were stained with a Calcein‐AM/PI Live/Dead Cell Double Stain Kit and then imaged under a fluorescence microscope.

### Evaluation of Type‐I Interferon (IFN‐I) Responses

MC38, 4T1, B16F10, or RAW264.7 cells were seeded in 6‐well plates at a density of 2 × 10^5^ cells per well. Following overnight incubation, the cells were treated with 5BThi, TACTICs, CS (CS: 60 µg mL^−1^), or ADU‐S100 (20 µg mL^−1^) for 24 h. The cells were washed and collected for RT‐qPCR analyses. For RT‐qPCR analyses, RNA was isolated using the following reagents: 0.25 mol L^−1^ NaCl, 0.05 mol L^−1^ Tris HCl (pH 7.5), 20 mmol L^−1^ EDTA, 1% SDS (M/V). All these reagents were dissolved in diethyl pyrocarbonate (DEPC) water, and sterilized at 121 °C for 20 min before use. Reverse transcription was performed using the HiScript lll RT SuperMix for qPCR (+gDNA wiper) (Vazyme, R323‐01). Quantitative real‐time PCR was performed with the Taq Pro universal SYBR qPCR Master Mix (Vazyme, Q712‐02) using a Bio‐Rad CFX Connect Real‐Time PCR Detection System. The expression of the target genes was normalized to the housekeeping genes *Hprt* using 2^−ΔΔCq^ method.

### Reactive Oxygen Species (ROS) Detection

MC38 or 4T1 cells were seeded in 6‐well plates at a density of 2 × 10^5^ cells per well. Following overnight incubation, the cells were treated with 5BThi, TACTIC, or CS (CS: 60 µg mL^−1^) and incubated for 48 h. The culture medium was removed and the cells were collected and stained with DCFH‐DA (Beyotime) in serum‐free medium (1:1000, v/v) at 37 °C for 20 min. Finally, the cells were washed for three times with PBS and analyzed by a BD FACSVerse flow cytometer.

### In Vitro Immunofluorescence of Damaged DNA

MC38 cells were seeded in 12‐well plates at a density of 2 × 10^5^ cells per well. Following overnight incubation, the cells were treated with 5BThi, TACTIC, or CS (CS: 60µg mL^−1^). After incubating for another 24 h, the cells were stained by a DNA Damage Assay Kit by γH2AX Immunofluorescence and imaged with a confocal laser scanning microscope.

### Evaluation of Calreticulin (CRT) Expression

MC38 and 4T1 cells were seeded in 12‐well plates at a density of 3 × 10^5^ cells per well and cultured overnight. The cells were irradiated with an external beam X‐ray at a dose of 12 Gy. Following irradiation, the culture medium was replaced, and the cells were treated with TACTIC (CS: 60 µg mL^−1^) for 24 h. After incubation, the supernatant was discarded, and the cells were collected and washed twice with PBS. Anti‐CD16/32 was added and incubated for 10 min. Subsequently, Calreticulin‐ER marker was added and incubated for 30–60 min, followed by a 30‐min incubation with Goat anti‐Rabbit IgG (H+L) Cross‐Adsorbed Secondary Antibody Alexa‐647. Finally, the cells were fixed with 1% paraformaldehyde and analyzed using a BD FACSVerse flow cytometer.

### Maturation and Antigen Uptake of BMDCs

BMDCs were seeded in 12‐well plates at a density of 1 × 10^6^ cells per well and treated with TACTIC or CS (CS: 3 µg mL^−1^). 8 h later, BMDCs were collected and incubated with anti‐CD16/32 for 10 min, followed by staining with anti‐CD11c‐FITC, anti‐CD80‐APC, anti‐CD86‐PE, anti‐MHCII‐PerCP/Cy5.5 for flow cytometry analyses.

Cy5‐labeled OVA was pre‐incubated with LPS (100 ng mL^−1^), CS, or TACTIC for ≈15 min. The weight ratio of CS or TACTIC and Cy5‐OVA was 3/1. BMDCs were seeded in 12‐well plates at a density of 1 × 10^6^ cells per well and treated with Cy5‐OVA or the pre‐complexation of CS/Cy5‐OVA or TACTIC/Cy5‐OVA. 6 h later, BMDCs were collected and stained with anti‐CD11c‐FITC for flow cytometry analyses.

### RNA‐Sequencing Analyses

BMDCs were seeded in 12‐well plates at a density of 3 × 10^6^ cells per well and treated with CS or TACTIC (CS: 3 µg mL^−1^). 24 h later, RNA was isolated from these cells and stored at −80 °C before analyses. RNA‐sequencing library construction and analyses were performed at Beijing Novogene. Heatmaps were generated and KEGG differential analyses were performed using the Novomagic platform.

### Retention and Distribution of TACTIC in Tumor Tissues

To synthesize Cy7‐labeled TACTIC, Cy7‐NHS DMSO solution was added dropwise into TACTIC aqueous solution (Cy7‐NHS: TACTIC = 1:25, wt/wt) and the mixture was stirred at room temperature for 24 h in dark. After dialysis against deionized water using dialysis tubing (MWCO: 3.5k Da), Cy7‐labeled TACTIC was obtained by lyophilization. To study the retention of TACTIC in tumors, 50 µL of Cy7‐labeled TACTIC (0.3 mg mL^−1^) was intratumorally injected into the MC38 tumor‐bearing mice. The mice were scanned with an in vivo imaging system (IVIS, Lumina, PE) at 10, 24, 48, and 72 h after injection.

Cy5‐labeled TACTIC was synthesized using a similar protocol to that of Cy7‐labeled TACTIC as mentioned above. To evaluate the distribution of TACTIC in tumor tissues, 50 µL of Cy5‐labeled TACTIC (0.3 mg mL^−1^) was intratumorally injected into the MC38 tumor‐bearing mice. 24 h later, the mice were euthanized and the tumor tissues, tumor‐draining lymph nodes (TDLNs), and major organs (heart, liver, spleen, lung, and kidney) were isolated and scanned with an IVIS. The tumor tissues were then dissociated into single‐cell suspensions, stained with Zombie NIR, and incubated with anti‐CD16/32 for 10 min. After washing, the cells were stained with anti‐CD45‐PerCP/Cy5.5, anti‐CD3‐FITC, anti‐CD11b‐FITC, and anti‐CD11c‐PE/Cy7 for flow cytometry analyses.

### Anti‐Cancer Studies

To study the effects of TACTIC on tumor growth, 50 µL of TACTIC solution (0.3 mg mL^−1^) was intratumorally injected into mice on days 0, 3, 6, and 9. To study the effects of TACTIC on the growth of irradiated tumors, an external beam X‐ray irradiation with a dose of 12 Gy was performed on the tumors on day 0.6 h later, 50 µL of TACTIC solution (0.3 mg mL^−1^) was intratumorally injected. Then, TACTIC solutions at the same dosages were injected into the tumors on days 3, 6, and 9 since the initiation of treatment. Tumor diameters were measured every three days and tumor volumes were calculated using a formula: tumor volume = (a × b^2^)/2, where a is the long diameter and b is the short diameter of tumors. All of the tumor‐bearing mice were euthanized when the tumor volume reached 1500 mm^3^.

### Flow Cytometry Analyses of Tumors, TDLNs, Blood Cells and Spleens

An external beam X‐ray irradiation with a dose of 12 Gy was performed on the MC38 colorectal tumors, and 50 µL of TACTIC solution (0.3 mg mL^−1^) was intratumorally injected on the same day and 3 days later. On day 12 since the initiation of treatment, tumor tissues, TDLNs, blood cells, and spleens were collected for analyses.

For tumors, the tumor tissues were dissociated into single‐cell suspensions, stained with Zombie NIR, and incubated with anti‐CD16/32. After washing, the cells were stained with anti‐CD45‐PerCP/Cy5.5 and anti‐CD11c‐FITC for flow cytometry analyses.

For TDLNs, the tissues were dissociated into single‐cell suspensions, stained with Zombie NIR, and incubated with anti‐CD16/32. After washing, the cells were stained with anti‐CD45‐PerCP/Cy5.5, anti‐CD3‐APC, anti‐CD4‐PE, anti‐CD8‐FITC, and anti‐CD69‐PE/Cy7 for flow cytometry analyses.

For blood cells, the red blood cells were lysed using ACK lysis buffer. Then, the cells were stained with Zombie NIR and incubated with anti‐CD16/32. After washing, the cells were divided for CD69 staining and IFN‐γ ‐staining separately. For CD69 evaluation, the cells were stained with anti‐CD45‐PerCP/Cy5.5, anti‐CD3‐APC, anti‐CD4‐PE, anti‐CD8‐FITC, and anti‐CD69‐PE/Cy7 for flow cytometry analyses. For IFN‐γ evaluation, the cells were stained with anti‐CD45‐PerCP/Cy5.5, anti‐CD3‐FITC, anti‐CD4‐PE, anti‐CD8‐PE/Cy7, anti‐IFN‐γ‐APC for flow cytometry analyses.

For spleens, the tissues were dissociated into single‐cell suspensions, and the red blood cells were lysed using ACK lysis buffer. Then, the cells were stained with Zombie NIR and incubated with anti‐CD16/32. After washing, the cells were divided for CD4 T cell staining and CD8 T cell staining for flow cytometry analyses separately. For CD4 T cells staining, the cells were stained with anti‐CD45‐PerCP/Cy5.5, anti‐CD3‐APC, anti‐CD4‐PE/Cy7, anti‐CD44‐FITC and anti‐CD62L‐PE for flow cytometry analyses. For CD8 T cells staining, the cells were stained with anti‐CD45‐PerCP/Cy5.5, anti‐CD3‐APC, anti‐CD8‐PE/Cy7, anti‐CD44‐FITC, and anti‐CD62L‐PE for flow cytometry analyses.

### Statistical Analysis

All statistical analyses were performed using Prism 8 (GraphPad Software). For comparisons between the two groups, an unpaired *t*‐test was used. One‐way ANOVA was used to analyze gene expression, flow cytometry, ELISA, and tumor growth curves both in vitro and in vivo (except for the two‐group comparisons). Survival curves were compared using a log‐rank test. Data are presented as mean ± SD with a minimum of 3 biological replicates. For all graphs, *p < 0.05; **p < 0.01; ***p < 0.001.

## Conflict of Interest

The authors declare no conflict of interest.

## Author Contributions

H.H., L.L., Y.Z., and Z.Z. conceived the project; H.H., L.L., Y.Z. J.J., L.C., and C.Y. performed the experiments and analyzed the data; Y.S. provided technical input on this project; H.H., Y.Z., and Z.Z. drafted the manuscript; Y.Z. and Z.Z. supervised the project.

## Supporting information



Supporting Information

## Data Availability

The data that support the findings of this study are available from the corresponding author upon reasonable request.
