# Supplementary material for: Tumor‐Adhesive Chitosan‐Derived Multi‐Immune Agonist Unleashes Strong and Durable Anti‐Cancer Immunity
Source: Adv Sci (Weinh). 2025 Feb 25;12(16):2414110. doi: 10.1002/advs.202414110 (PMC12021070; doi:10.1002/advs.202414110)
Supplement: Supplementary file 1 — Supporting Information [file ADVS-12-2414110-s001.docx]

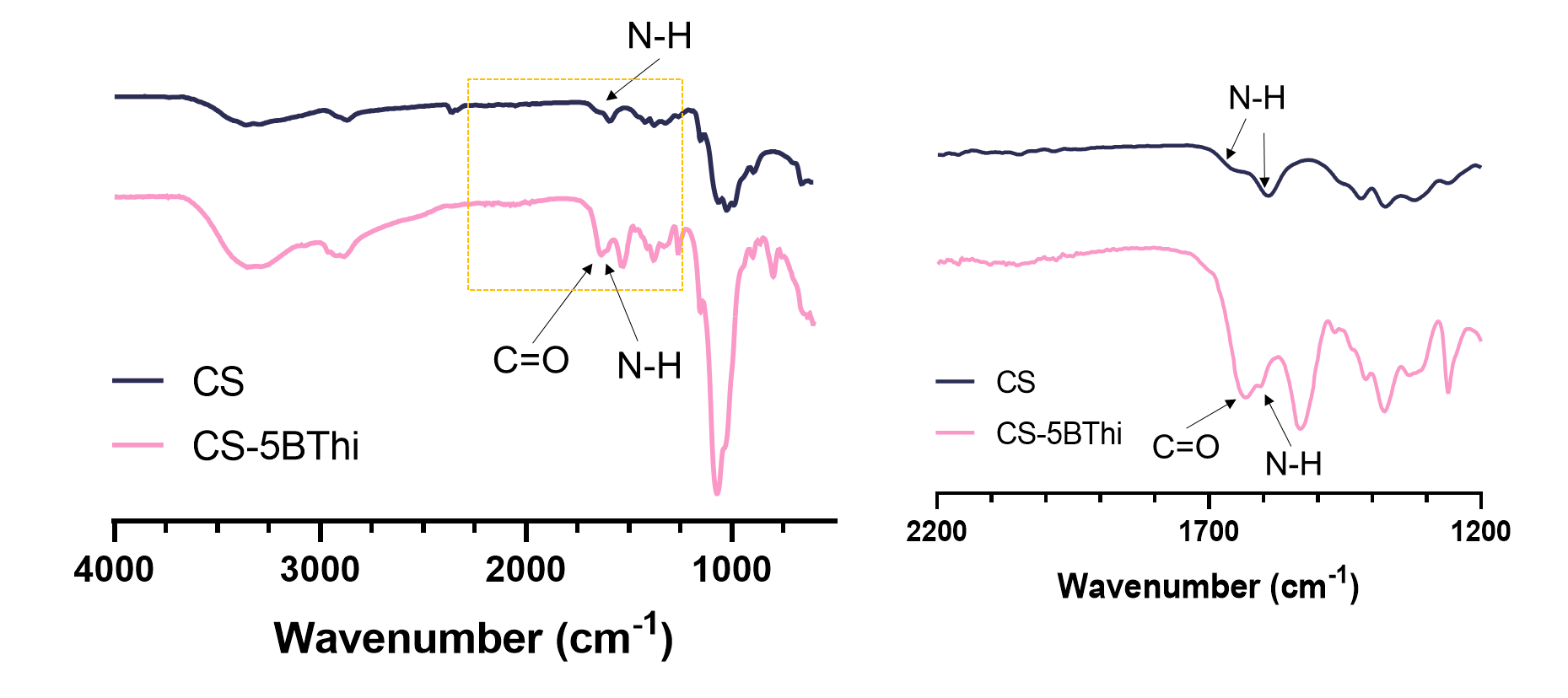


**Figure S1.** The FT-IR spectrum of CS and CS-5BThi TACTIC.


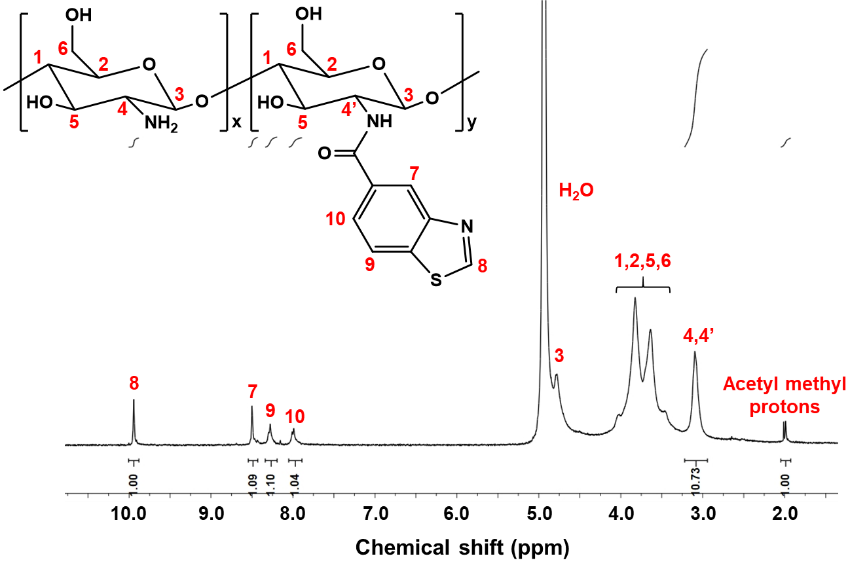


**Figure S2.** The ^1^H NMR spectrum of CS-5BThi TACTIC.


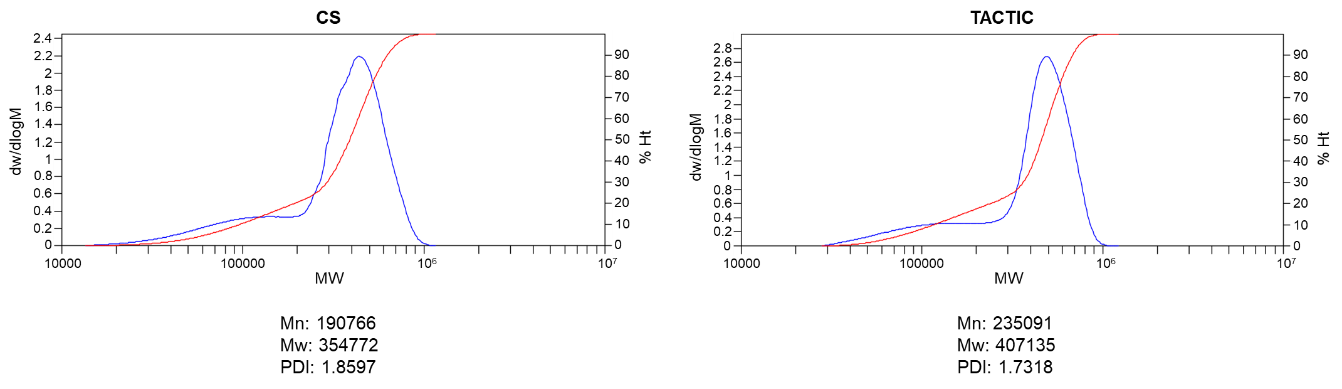


**Figure S3.** Gel permeation chromatography (GPC) characterization of CS and CS-5BThi TACTIC.


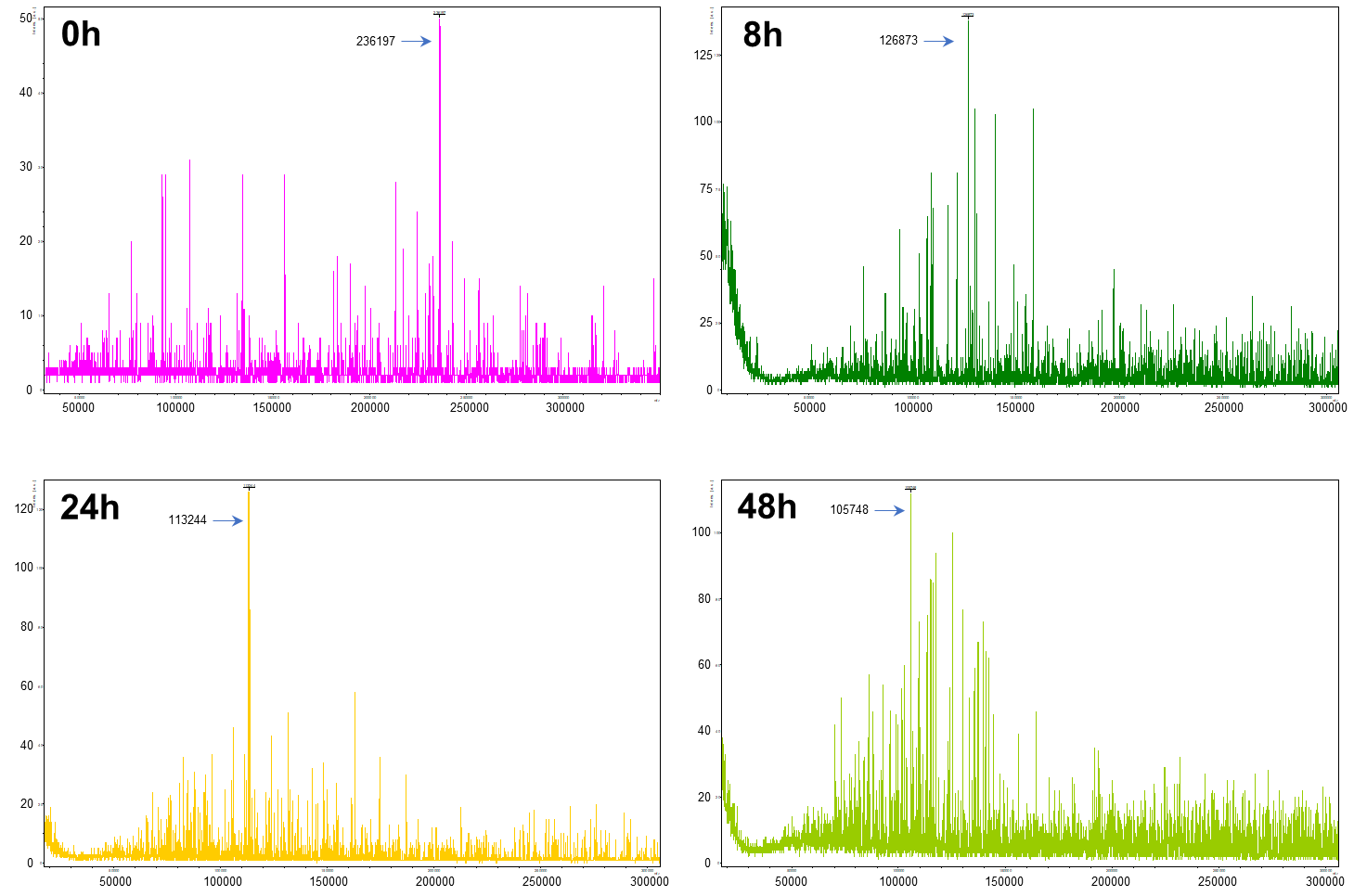


**Figure S4.** Mass spectrum analysis of CS-5BThi TACTIC after treated with 1 mg/mL lysozyme.

**Figure S5.** Zeta potential of CS and CS-5BThi TACTIC in phosphate buffer (pH 6.0).


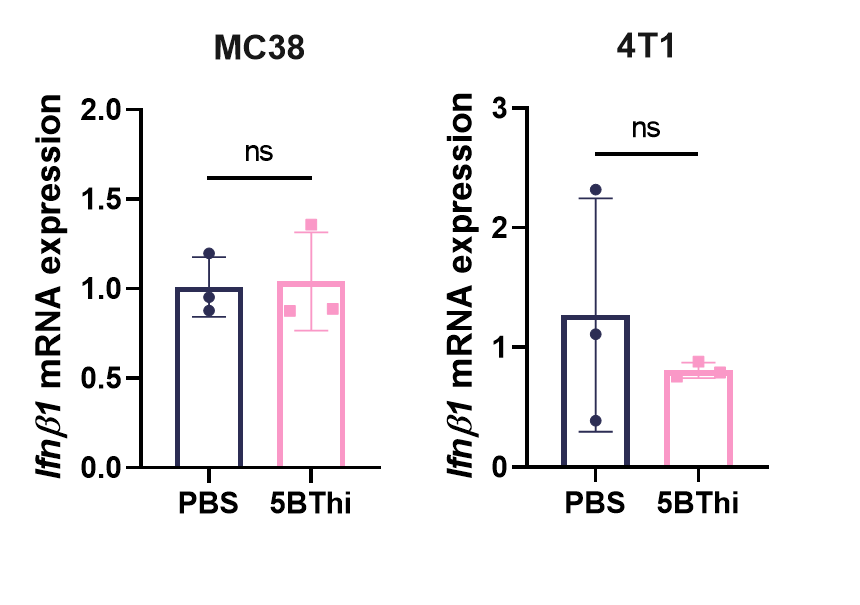


**Figure S6.** The relative mRNA expression of *Ifnβ1* in MC38 cells and 4T1 cells after treatment with 5BThi (n=3).


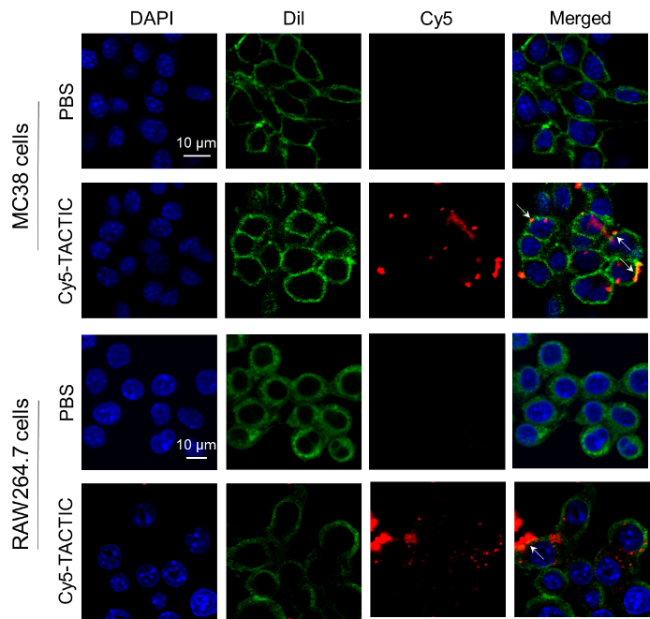


**Figure S7**. Confocal laser scanning microscope (CLSM) images of MC38 cells and RAW264.7 macrophages after treated with Cy5-labeled TACTIC for 24 hours. (Cy5 was denoted in red and Dil was denoted in green)


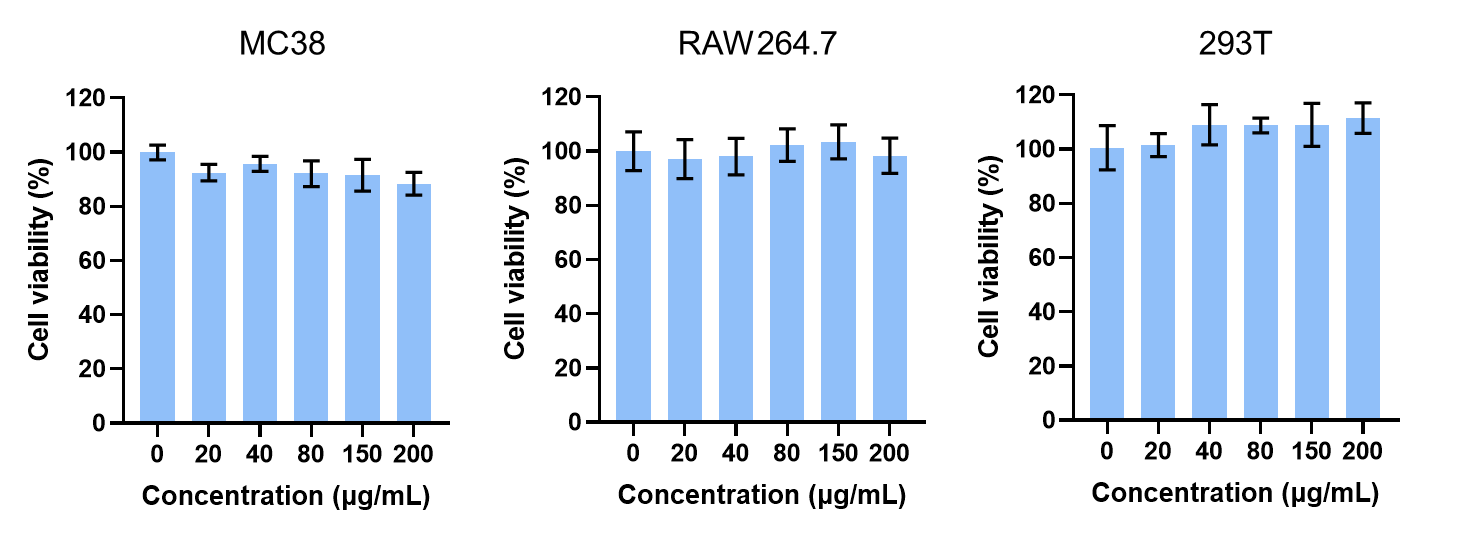


**Figure S8.** The cell viability of MC38 colorectal cancer, RAW264.7 macrophages and 293T cells after treatment with TACTIC at different concentrations (n=5).


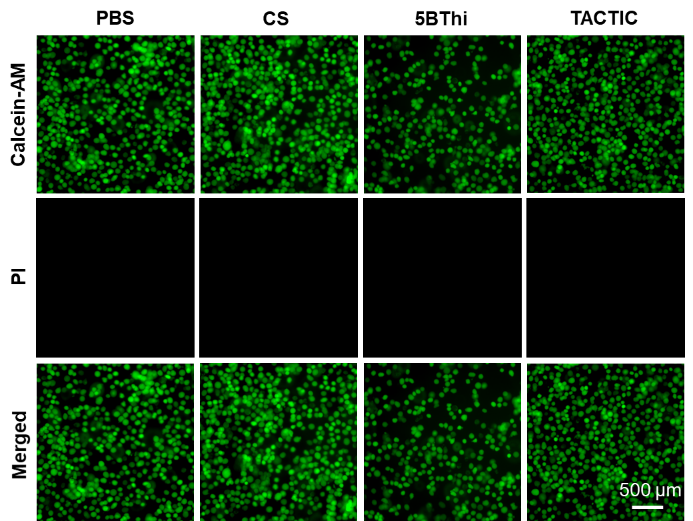


**Figure S9.** Fluorescence images of Calcein-AM (green, live cells) and PI (red, dead cells) co-stained MC38 cells at 24h after treated with chitosan, 5BThi or TACTIC.


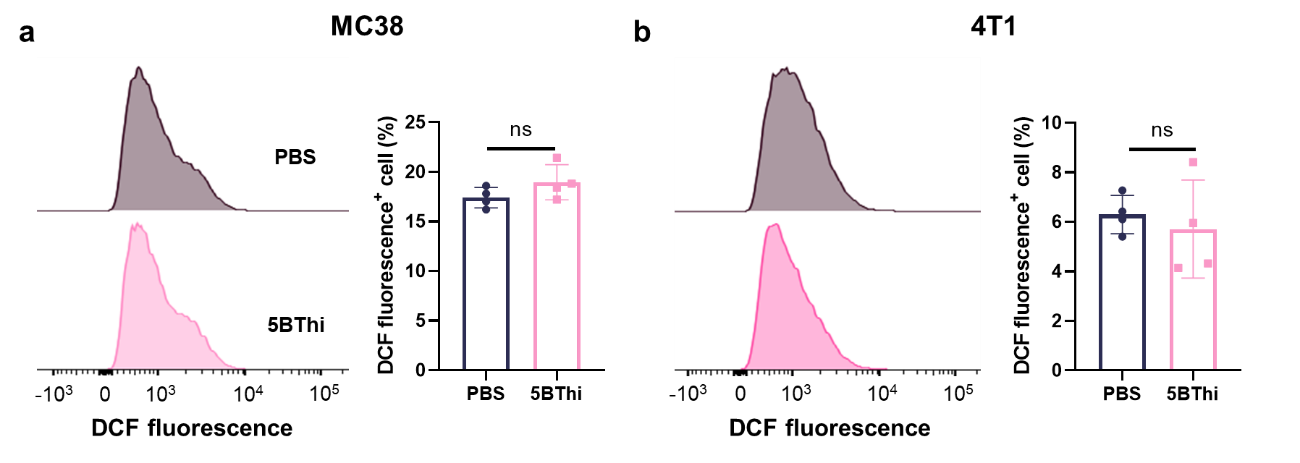


**Figure S10.** Histogram graphs of DCF fluorescence and the percentages of DCF fluorescence positive cells in **(a)** MC38 cells and **(b)** 4T1 cells after treatment with 5BThi (n=4).


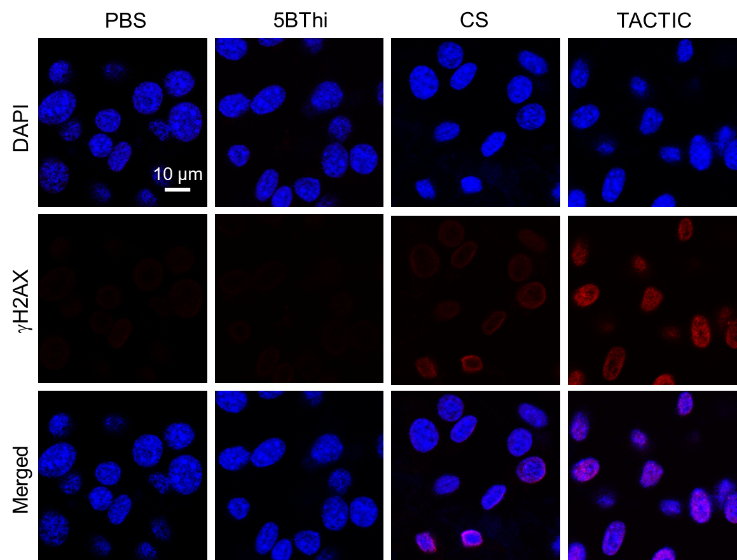


**Figure S11.** The fluorescence images indicated the cell nucleus (DAPI) and damaged DNA (γH2AX) of MC38 cells after treatment with 5BThi, CS or CS-5BThi TACTIC.

**Figure S12.** The concentration of lactate in the supernatant of 4T1 cells after treatment with CS or TACTIC (n=3).


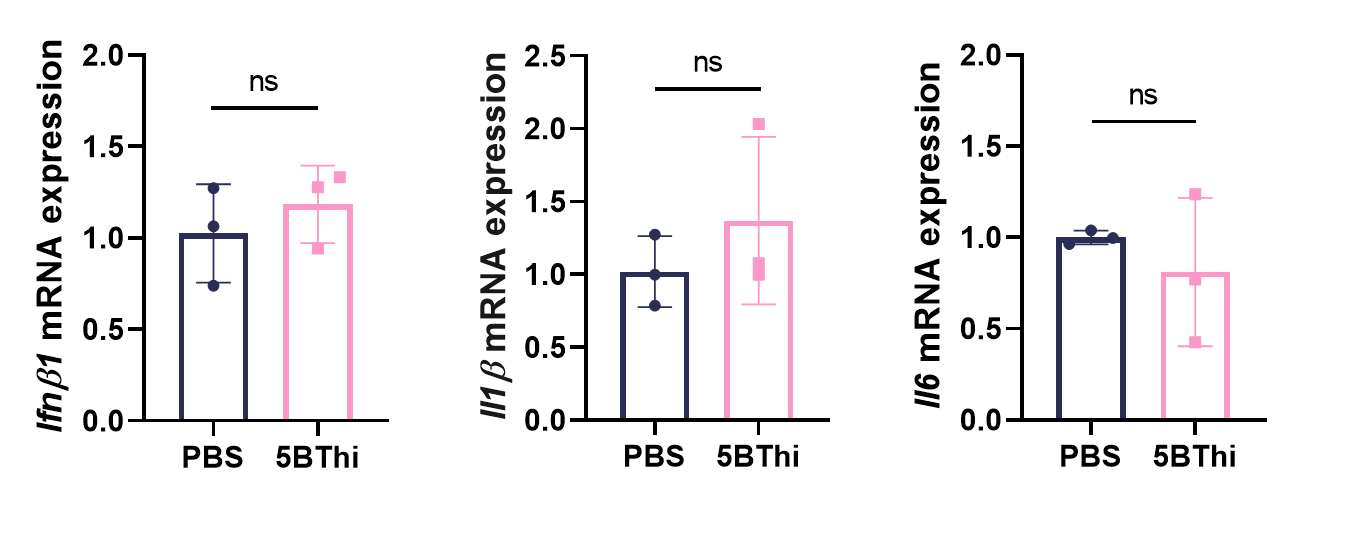


**Figure S13**. The relative mRNA expression of *Ifnβ1*, *Il1β* and *Il6* in RAW264.7 macrophages after treatment with 5BThi (n=3).


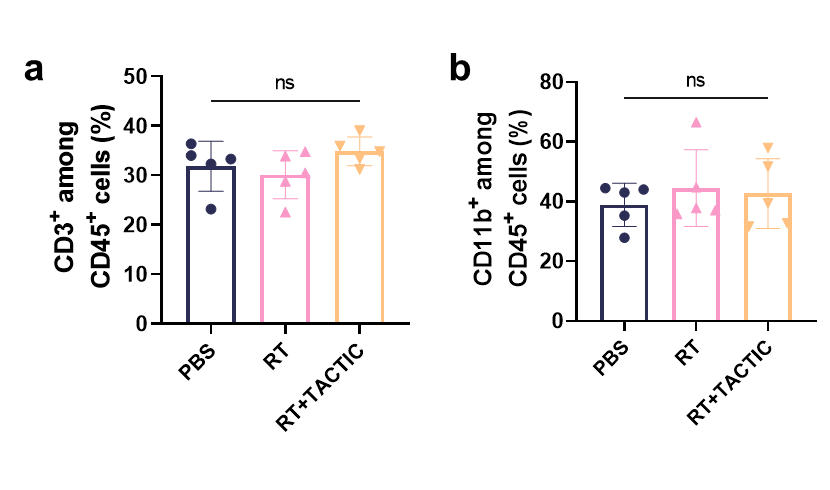


**Figure S14.** Percentages of **(a)** CD3^+^ T cells among CD45^+^ cells and **(b)** CD11b^+^ myeloid cells among CD45^+^ cells in tumor tissues at day 12 since the initiation of treatment (n=5).


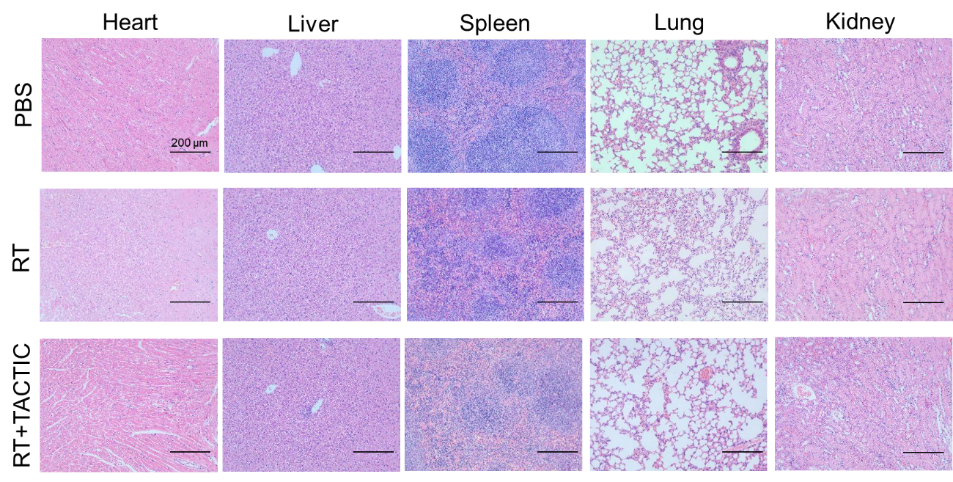


**Figure S15**. The representative H&E images of the organs in the MC38 colorectal cancer-bearing mice after the indicated treatment. The treatments were performed per **Figure 6a**.

**Table S1.** Grafting rates of azole molecules on different TACTICs determined using UV spectroscopy.

| TACTIC | Grafting percentages |
| --- | --- |
| CS-4BImi TACTIC | 11.82 |
| CS-5BImi TACTIC | 12.96 |
| CS-5BThi TACTIC | 9.41 |
| CS-4Ind TACTIC | 10.10 |
| CS-5Ind TACTIC | 14.51 |
| CS-6Ind TACTIC | 11.22 |

**Table S2.** The primer sequences used for RT-qPCR.

| Gene | Forward Primer | Reverse Primer |
| --- | --- | --- |
| *Hprt* (mouse) | AGCCTAAGATGAGCGCAAGT | GGCCCACAGGACTAGAACACC |
| *Ifnβ* (mouse) | TCCACCAGCAGACAGTGTTTC | TCAAGTGGAGAGCAGTTGAGG |
| *Il6* (mouse) | AACGATGATGCACTTGCAGA | GAGCATTGGAAATTGGGGTA |
| *Il1β* (mouse) | TGATTCAAGGGGACATTAGGCA | ACCAATTCATCCCCCACACG |

**Table S3.** The antibodies used for flow cytometry.

| Antibody/Marker | Fluorophore | Clone | Dilution | Company | Cat number |
| --- | --- | --- | --- | --- | --- |
| Live/Dead | Zombie NIR™ |  | 1:100 | Biolegend | 423106 |
| CD16/32 |  |  | 1:50 | Biolegend | A2110 |
| CD11c | FITC | N418 | 1:200 | Biolegend | 117306 |
| CD11b | FITC | M1/70 | 1:100 | Biolegend | 101206 |
| CD3 | FITC | 17A2 | 1:100 | Biolegend | 100204 |
| CD3 | APC | 17A2 | 1:200 | Biolegend | 100236 |
| CD4 | PE | GK1.5 | 1:200 | Biolegend | 100408 |
| CD4 | PE/Cy7 | GK1.5 | 1:400 | Biolegend | 100422 |
| CD8a | FITC | 53-6.7 | 1:100 | Biolegend | 100706 |
| CD8a | PE/Cy7 | 53-6.7 | 1:400 | Biolegend | 100722 |
| CD44 | FITC | IM7 | 1:200 | Biolegend | 103006 |
| CD45 | PerCP/Cy5.5 | 30-F11 | 1:400 | Biolegend | 103132 |
| CD62L | PE | MEL-14 | 1:200 | Biolegend | 104408 |
| CD69 | PE/Cy7 | H1.2F3 | 1:200 | Biolegend | 104511 |
| CD80 | APC | 16-10A1 | 1:400 | Biolegend | 104714 |
| CD86 | PE | GL1 | 1:400 | Thermofisher | 12-0861-83 |
| I-A/I-E (MHCII) | PerCP/Cy5.5 | M5/114.15.2 | 1:400 | Thermofisher | 107626 |
| IFNγ | APC | XMG1.2 | 1:200 | Biolegend | 505810 |
| Calreticulin-ER Marker |  |  | 1:1000 | Abcam | 1030675-8 |
| Goat anti-Rabbit IgG (H+L) Cross-Adsorbed Secondary Antibody | Alexa 647 |  | 1:600 | ThermoFisher | 2527970 |
